# Standardized Ethanol Extract of *Cassia mimosoides* var. *nomame* Makino Ameliorates Obesity via Regulation of Adipogenesis and Lipogenesis in 3T3-L1 Cells and High-Fat Diet-Induced Obese Mice

**DOI:** 10.3390/nu15030613

**Published:** 2023-01-25

**Authors:** So-Won Heo, Kyung-Sook Chung, Young-Seo Yoon, Soo-Yeon Kim, Hye-Shin Ahn, Yu-Kyong Shin, Sun-Hee Lee, Kyung-Tae Lee

**Affiliations:** 1 Department of Pharmaceutical Biochemistry, College of Pharmacy, Kyung Hee University, 26 Kyungheedae-ro, Seoul 02447, Republic of Korea; 2 Department of Biomedical and Pharmaceutical Science, Graduate School, Kyung Hee University, 26 Kyungheedae-ro, Seoul 02447, Republic of Korea; 3 Department of Fundamental Pharmaceutical Science, Graduate School, Kyung Hee University, 26 Kyungheedae-ro, Seoul 02447, Republic of Korea; 4 Department of New Material Development, COSMAXBIO, Seongnam 13486, Republic of Korea

**Keywords:** *Cassia mimosoides*, high-fat diet, AMPK, adipogenesis, lipogenesis, white adipose tissue, brown adipose tissue, microbiota

## Abstract

Obesity is a major cause of conditions such as type 2 diabetes and non-alcoholic fatty liver disease, posing a threat to public health worldwide. Here, we analyzed the anti-obesity effects of a standardized ethanol extract of *Cassia mimosoides* var. *nomame* Makino (EECM) in vitro and in vivo. Treatment of 3T3-L1 adipocytes with EECM suppressed adipogenesis and lipogenesis via the AMP-activated protein kinase pathway by downregulating the expression levels of CCAAT/enhancer-binding protein-alpha, peroxisome proliferator-activated receptor (PPAR)-γ, sterol regulatory element-binding protein-1, and fatty acid synthase and upregulating the acetyl-CoA carboxylase. EECM inhibited mitotic clonal expansion during early adipocyte differentiation. Oral administration of EECM for 10 weeks significantly alleviated body weight gain and body fat accumulation in high-fat diet (HFD)-fed mice. EECM mitigated adipogenesis and lipid accumulation in white adipose and liver tissues of HFD-induced obese mice. It regulated the levels of adipogenic hormones including insulin, leptin, and adipokine in the blood plasma. In brown adipose tissue, EECM induced the expression of thermogenic factors such as uncoupling protein-1, PPAR-α, PPARγ co-activator-1α, sirtuin 1, and cytochrome c oxidase IV. EECM restored the gut microbiome composition at the phylum level and alleviated dysbiosis. Therefore, EECM may be used as a promising therapeutic agent for the prevention of obesity.

## 1. Introduction

Incidence of obesity and overweight is very high worldwide, and recent statistics estimate that more than 1.9 billion adults are overweight worldwide, of which more than 650 million adults develop obesity. As the prevalence of obesity increases, the risk of various diseases and complications such as type 2 diabetes (T2D), non-alcoholic fatty liver disease (NAFLD), hypertension, cardiovascular disease (CVD), insulin resistance, and some types of cancers also increases [1,2,3]. Obesity ultimately results in hypertrophy and hyperplasia of adipocytes and adipogenesis via an imbalance between energy intake and expenditure [4]. Regulation of adipogenesis may be an effective therapeutic strategy for treating obesity. Therefore, it is important to identify potential adipogenesis-regulating anti-obesity drugs.

Mitotic clonal expansion (MCE) is an essential process during the early differentiation of adipocytes. After the proliferation of adipocytes, MCE is initiated, which induces intracellular lipid accumulation, resulting in an adipocyte phenotype [5]. Adipogenesis and lipogenesis are modulated mainly by AMP-activated protein kinase (AMPK), a major regulator of the cellular energy balance pathway [6]. AMPK activity suppresses the expression of crucial adipogenic transcription factors, including CCAAT/enhancer-binding protein alpha (C/EBPα), peroxisome proliferator-activated receptor gamma (PPARγ), and sterol regulatory element-binding protein (SREBP)-1 [7]. In addition, AMPK regulates the expression of lipolysis and lipogenesis proteins such as acetyl-CoA carboxylase (ACC) and fatty acid synthase (FAS) [8]. Therefore, inhibition of adipogenic and lipogenic signaling of adipocytes via the AMPK pathway may potentially alleviate obesity.

Body weight gain is often induced by white adipose tissue (WAT), which stores nutrient-derived energy [9]. In contrast, brown adipose tissue (BAT) plays an important role in energy expenditure via thermogenesis and is composed of more mitochondria than WAT [10]. Thermogenesis in BAT is induced by the upregulation of mitochondrial factors, such as uncoupling protein-1 (UCP-1), cytochrome c oxidase (COX)-IV, and thermogenic proteins, such as PPARγ co-activator-1α (PGC-1α), sirtuin 1 (SIRT1), and PPARα. Interestingly, the activation of thermogenesis in BAT appears to inhibit body weight gain [11] and diabetes status [12].

In human intestine, the gut microbiota consists of various bacterial phyla such as Firmicutes, Bacteroidetes, Proteobacteria, Actinobacteria, and Verrucomicrobia. Moreover, Firmicutes and Bacteroidetes, two dominant phyla in the gut microbiota, constitute more than 90% of the total bacterial community [13]. The Firmicutes-to-Bacteroidetes (F/B) ratio has a critical effect on the maintenance of homeostasis in the intestinal environment. Interestingly, a rapid change in the F/B ratio can lead to intestinal microbial imbalance, called dysbiosis, which correlates with obesity [14]. Therefore, it is important to develop novel anti-obesity drugs and evaluate their effects on the gut microbiome.

Various synthetic drugs exert anti-obesity effects, but their application is limited by certain side effects, such as diarrhea, CVD, and dizziness. Therefore, many studies have focused on developing anti-obesity drugs based on natural products with relatively low side effects. *Cassia mimosoides* var. *nomame* Makino grows in most regions of Korea and is also distributed in Japan and China. It is used in herbal tea, and its fruit is used for medicinal purposes. Previous studies have reported that the 10% methanol extract of *C. mimosoides* var. *nomame* Makino attenuates myocardial injury [15] and brain damage [16] by inhibiting apoptosis. However, the anti-obesity effects of *C. mimosoides* var. *nomame* Makino and its molecular signaling pathway have not yet been reported. Therefore, we investigated the effects of the 30% ethanol extract of *C. mimosoides* var. *nomame* Makino (EECM) on obesity and clarified the underlying mechanisms in 3T3-L1 cells and high-fat diet (HFD)-induced obese animal models.

## 2. Materials and Methods

### 2.1. EECM Preparation, Identification, and Standardization

The leaves of *C. mimosoides* var. *nomame* Makino were collected from Youngwolgun (Gangwon-do, Republic of Korea). *C. mimosoides* var. *nomame* Makino was identified by miDNA Genome Research Institute (Gunsan, Jeollabuk-do, Republic of Korea). A voucher specimen (COS2007) was stored and extracted according to the instruction of COSMAX BIO (Seongnam, Gyeonggi-do, Republic of Korea). After 30% ethanol extraction, the yield of 30% ethanol extract of *C. mimosoides* var. *nomame* Makino (EECM) was 19.66% (dried leaves of *C. mimosoides* var. *nomame* Makino; 1.0 kg, extract residue; 196.6 g). Ultra-performance liquid chromatography (UPLC) analysis was performed with an Agilent 6545 Q-TOF LC/MS spectrometer (Agilent Technologies, Santa Clara, CA, USA). The chromatographic separation was achieved on EclipsePlus C18 (1.8 μm, 50 × 2.1 mm, Agilent Technologies) and column temperature at 35 °C. The mobile phase consisted of acetonitrile (solvent A) and 0.1% formic acid in water (solvent B). The gradient condition of the mobile phase was 0–10 min, 10% to 100%; 10–15 min, 100% to 100%; 15–20 min, 100% to 10%, as a percentage of solvent A. The flow rate was set at 0.7 mL/min, and the injection volume was 1.0 µL. The monitored wavelength of the UV detector was 210, 254, and 315 nm. The conditions of MS analysis in the positive or negative ion mode were as follows: mass range: 100–1700 m/z; acquisition rate: 1 spectra/s; acquisition time: 100 ms/spectrum; gas temperature: 320 °C; drying gas: 8 L/min; nebulizer: 35 psi; sheath gas temperature: 350 °C; sheath gas flow: 11 L/min; Vcap: 3500 V; MS fragmentor: 100 V. The gradient mobile phases was set up as follows: solvent A: 100% acetonitrile and solvent B: distilled water with a gradient elution as follows: 0 to 40 min, 10% to 30%; 40 to 50 min, 30% to 100%; 50 to 55 min, 100% to 100%; 55 to 60 min, 100% to 10% as a percent of solvent A at a flow rate of 1.0 mL/min.

### 2.2. Differentiation of Adipocytes

The cell culture of 3T3-L1 mouse preadipocyte and differentiation was performed as in our previous report [7]. During the differentiation of 3T3-L1 mouse preadipocyte, the media were changed to DM containing 1 μM insulin with or without EECM (6.25, 12.5, or 25 μg/mL) every 2 days and processed repeatedly for 7 days.

### 2.3. Cell Viability

Cells were cultured at 1 × 10^5^ cells/mL into 24-well plates with GM for 3 days. Then, the medium was changed to DM including MDI with or without EECM (6.25, 12.5, or 25 μg/mL) and the treatment of EECM until 7 days. To examine cell viability of the EECM, the tetrazolium compound [3-(4, 5-dimethylthiazol-2-yl)-5-(3-carboxymethoxyphenyl)-2-(4-sulfophenyl)-2H-tetrazolium, inner salt; MTS] solution (Promega Corporation, Madison, WI, USA) was treated with 10% of the media volume in each well and then incubated for 2 h. After MTS treatment, the measurement of absorbance was performed at 490 nm.

### 2.4. Oil Red O Staining

After the differentiation of 3T3-L1 cells, the cells were then fixed with 4% formaldehyde in PBS for 40 min at 25 °C. 0.2% Oil Red O in isopropanol was diluted with DW and filtered using filter paper. The fixed cells were dried completely and then stained with Oil Red O solution for 1 h in dark conditions. The stained intracellular lipid droplets were observed with a microscope (OLYMPUS CORPORATION, Tokyo, Japan). In addition, the intracellular lipid droplets were liquified with 100% isopropanol and quantified by measuring the absorbance at 510 nm.

### 2.5. Western Blot Analysis

Protein lysates were prepared by PRO-PREP™ solution (Intron Biotechnology, Seoul, Korea) from cells or tissues. The lysate was reacted on ice, centrifuged at 13,000× *g* rpm at 4 °C, and the protein-contained supernatant was obtained. After the quantification of proteins (30 μg) using the Bradford assay, proteins were separated by SDS-PAGE and then transferred onto the PVDF membrane. The PVDF membranes were treated with the primary antibodies in skim milk solution for 18 h at 4 °C. The immunoblotted membranes were reacted with the secondary antibodies in skim milk solution at 25 °C and then detected by our previous report [7].

### 2.6. MCE Determination

For the analysis of MCE, propidium iodide (PI) staining was implemented. The 3T3-L1 cells were cultured with or without EECM treatment for 24 h and harvested. Subsequently, the cells were resuspended in PBS and fixed for 18 h in 70% cold ethanol at −20 °C. After fixation, the cells were stained with 100 μg/mL PI staining buffer containing RNase for 30 min in dark conditions. PI stained cells were analyzed by our previous report [17].

### 2.7. HFD-Induced Obese Animals Experiments

C57BL/6J mice (male, 5–6 weeks, 20–22 g) were purchased from Orient Bio Inc. (Seongnam, Republic of Korea) and cared for under standard conditions (light–dark cycle: 12 h, temperature: 22 ± 1 °C, humidity 40–60%) for 7 days. Mice were classified into 5 groups (*n* = 8/group) to inspect the anti-obesity effect of EECM: (i) control (CON) group, (ii) 60% HFD-induced obese group, (iii) HFD + orlistat administration group, (iv) HFD + EECM administration group (EECM 100 mg/kg, p.o.), and (v) HFD + EECM administration group (EECM 300 mg/kg, p.o.). The CON group was fed a normal diet, and the other group was fed a 60% HFD to induce obesity for 10 weeks. Simultaneously with the diet feeding, the CON and HFD groups were administered with vehicle, and orlistat and EECM groups were administered orlistat (20 mg/kg, p.o.) and EECM (100 or 300 mg/kg, p.o.), respectively. All administration of samples was daily provided to experimental animals. The body weight was measured 2 days per week. At the end of the experiment, sacrifice was performed surgically, and obtained tissues were immediately stored at −80 °C.

### 2.8. Body Fat Composition Analysis

The body fat composition of experimental animals was determined using dual-energy X-ray absorptiometry (DEXA) (InAlyzer, Medikors, Seongnam, Republic of Korea). The lean tissue, fat tissue, and differentiated tissue from lean to fat were exposed as blue, red, and green/yellow, respectively.

### 2.9. Hematoxylin and Eosin (H&E) Staining Analysis

White adipose tissue (WAT) and liver were obtained after mice sacrifice and then fixed with 4% formaldehyde in PBS buffer for 24 h. Fixed tissues were sliced and embedded into a paraffin block. H&E staining was performed, and then, stained tissue was observed with a light microscope optically, and the adipocyte size was measured by using an instrument of the microscope system (cell Sense standard ver.1.9, OLYMPUS CORPORATION, Tokyo, Japan).

### 2.10. Biochemical Examination of Blood Plasma

At the sacrifice, the blood of mice was collected from a vein and placed in heparin-coated tubes. The preparation and biochemical examination of plasma were followed by the guideline of T&P Bio Co. (Gwangju, Republic of Korea).

### 2.11. Genomic DNA Extraction and Microbiome Taxonomic Profiling (MTP)

Total genomic DNA (gDNA) from stool was extracted with the QIAamp^®^ Fast DNA Stool Mini Kit (Qiagen, Hilden, Germany) as the instruction of manufacture. Then, the bacterial 16S ribosomal RNA V3-V4 region was amplified by PCR. The amplified product was performed purification by magnetic bead reaction and detected by electrophoresis on agarose gel (1.5%). Whole products were pooled at a constant concentration to produce a library. The library was quantified at 2 nM using KAPA Library Quantification Kit Illumina^®^ platforms (Roche, Basel, Switzerland), and the sequence was analyzed using the Illumina iSeq 100 sequencing system (Illumina, San Diego, CA, USA) finally.

### 2.12. Statistical Analysis

Statistical values were calculated by the Prism Graph Pad 5.0 program (GraphPad Software Inc., San Diego, CA, USA). All data were expressed as mean ± SD for in vitro experiments and SEM for in vivo experiments. The analysis was performed by Tukey’s range test. *p*-values of less than 0.05 were considered statistically significant.

## 3. Results

### 3.1. Identification of Quercitrin and Standardization of EECM via UPLC–High-Resolution Mass Spectrometry (HR/MS)

**Figure 1:**
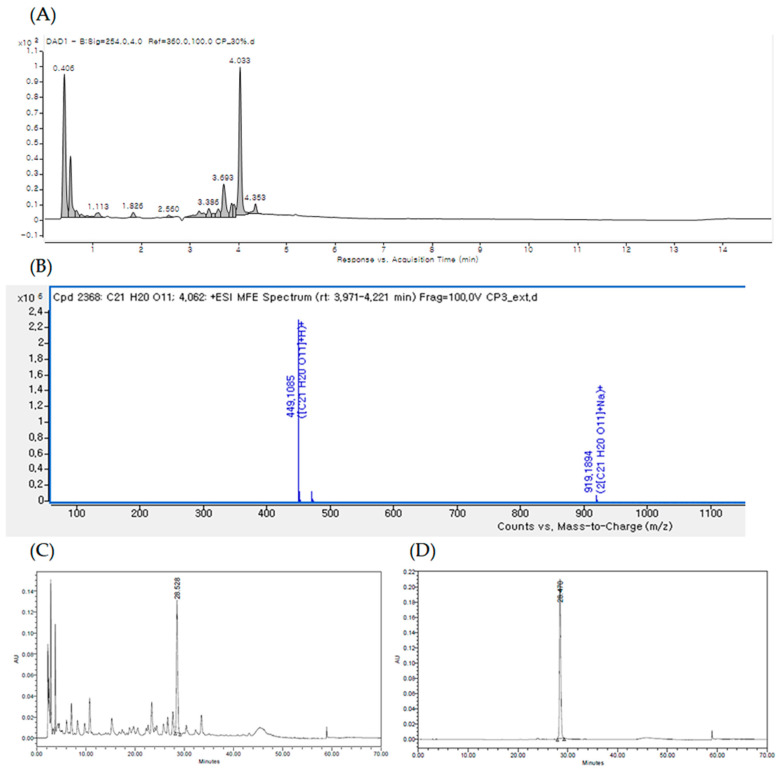
(**A**) UPLC of the ethanol extract of *C. mimosoides* var. *nomame* Makino (EECM) detected at 254 nm. (**B**) High-resolution mass spectrometry (HR/MS) spectrum of [M + H] ions (m/z 499) of quercitrin (Rt: 4.033 min). High-performance liquid chromatography (HPLC) profiles of (**C**) EECM and (**D**) standard quercitrin.

The UPLC chromatogram of EECM at 254 nm is shown in Figure 1A. By comparing the retention time, MS data, and UV/vis spectra with those presented in previous literature reports, the major peak at a retention time of 4.033 min [M + H] ions (m/z 499) was identified as quercitrin (Figure 1A,B). Qualitative analysis confirmed quercitrin by comparing the HPLC retention time data with the analytical standard of quercitrin (phytolab, GmbH & Co., Germany; Figure 1C,D).

### 3.2. EECM Inhibits Differentiation and Regulates Lipogenesis and Adipogenesis via AMPK Pathway in 3T3-L1 Preadipocytes

**Figure 2:**
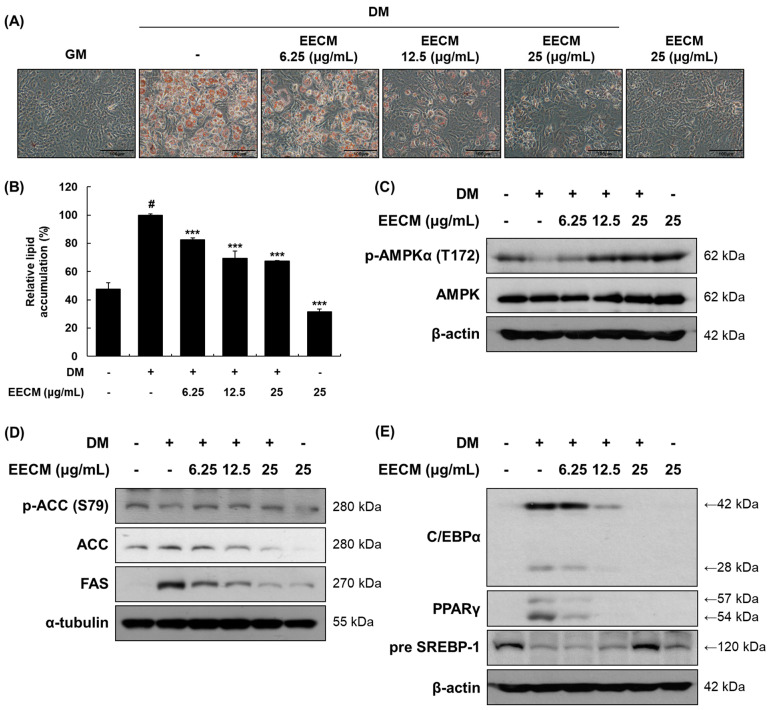
Inhibitory effects of EECM on intracellular lipid accumulation, adipogenesis, and lipogenesis in 3T3-L1 cells. The 3T3-L1 cells were cultured in the growth medium (GM) for four days. After the cells reached confluency, they were cultured with or without methylisobutylxanthine, dexamethasone, and insulin (MDI) and various concentrations of EECM. (**A**) Relative microscopic images of Oil-Red-O-stained 3T3-L1 cells. (**B**) Quantitative lipid accumulation ratio. After differentiation, total protein was extracted and detected by via Western blotting analysis. Expression levels of (**C**) AMPKα pathway proteins and (**D**) lipogenic and (**E**) adipogenic transcription factors. Values are represented as the mean ± standard deviation (SD). ^#^ *p* < 0.05 vs. the GM group; *** *p* < 0.001 vs. the differentiation medium (DM) group.

To investigate the effects of EECM on 3T3-L1 preadipocytes, we first performed the MTS assay at various concentrations (Figure S1). Cell viability was shown to be less than 80% with EECM concentration of 50 or 100 μg/mL, so we used 6.25 to 25 μg/mL of EECM for further experiments. After differentiation of 3T3-L1 cells with EECM, cell viability was shown to be more than 80% at all concentrations up to 25 μg/mL. Thus, we performed the following experiments with EECM concentrations of 6.25–25 μg/mL. We assessed the effect of EECM on intracellular lipid accumulation via Oil Red O staining. EECM visually and quantitatively inhibited lipid accumulation during investigation of the differentiated 3T3-L1 cells (Figure 2A,B). Next, we examined the regulation of EECM on the protein expression levels of phosphorylated AMPKα, lipogenic enzymes including ACC and FAS, and adipogenic transcription factors including C/EBPα, PPARγ, and SREBP-1. Phosphorylation of AMPKα (Thr172) decreased with MDI treatment and was restored by EECM (Figure 2C). Moreover, EECM increased the expression levels of phospho-ACC (Ser79) and pre-SREBP-1 and reduced the expression of total ACC, FAS, C/EBPα, and PPARγ in the differentiated cells (Figure 2D,E). Our data revealed that EECM disturbs lipogenesis and adipogenesis during adipocyte differentiation by regulating the AMPK pathway.

### 3.3. EECM Suppresses MCE during Early Differentiation of 3T3-L1 Preadipocytes

**Figure 3:**
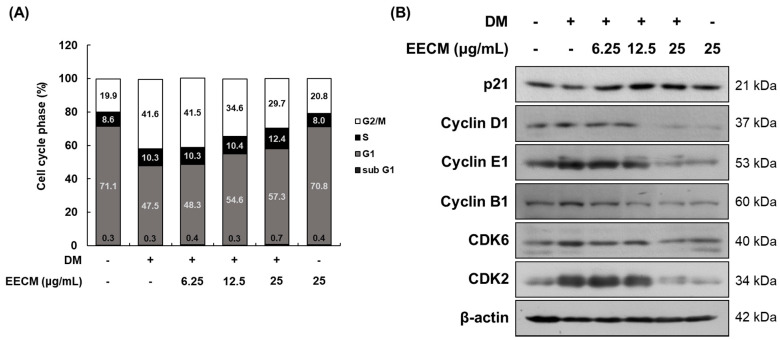
Regulatory effect of EECM on the MCE in 3T3-L1 cells. Differentiated cells were treated with EECM (6.25, 12.5, and 25 μg/mL) for 24 h. (**A**) Cell cycle phase ratio and (**B**) cell-cycle-related proteins expression were measured via propidium iodide (PI) staining and Western blotting, respectively.

When the cells are differentiated by MDI treatment, MCE progresses to the early stage. To demonstrate the inhibitory effect of EECM on MCE, we conducted cell cycle analysis after propidium iodide (PI) staining using flow cytometry. In Figure 3A, which shows 3T3-L1 preadipocytes differentiation with MDI, the G0/G1 phase ratio decreased considerably (from 71.1 to 47.5%), and the G2/M phase ratio increased accordingly (from 19.9 to 41.6%). The changes in this ratio suggested that the cell differentiation was initiated by MDI treatment, and MCE was successfully induced. In contrast, EECM induced an increase in the G0/G1 phase ratio compared to GM treatment (from 47.5 to 48.3, 54.6, and 57.3%, respectively) while reducing the G2/M phase ratio (from 41.6 to 41.5, 34.6, and 29.7%, respectively) in a concentration-dependent manner. Additionally, we investigated the protein expression levels of cell-cycle-related factors following EECM treatment in preadipocytes via Western blotting analysis. Expression levels of p21, a cell cycle inhibitor, decreased but showed a tendency to increase after EECM treatment. In contrast, the expression levels of cyclin D1, cyclin E1, cyclin B1, CDK6, and CDK2, indicating cell-cycle-related regulators, were increased after MDI treatment compared to those observed after GM treatment, but treatment with EECM, especially at high concentrations (25 μg/mL), markedly decreased the expression levels during the differentiation of adipocytes (Figure 3B).

### 3.4. EECM Obstructs the Body Weight Gain of HFD-Induced Obese Mice

**Figure 4:**
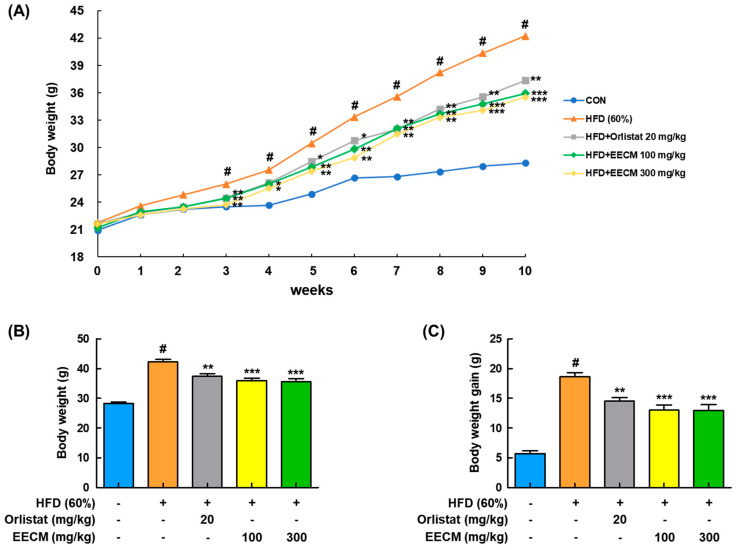
Repressive effect of EECM on body weight gain in HFD (60%)-induced obese mice. Mice of EECM treatment were provided the indicated dose of EECM (100 and 300 mg/kg) daily during the experiment. (**A**) Body weight changes, (**B**) total body weight at the endpoint of the experiment, and (**C**) total body weight gain for 10 weeks. Values are represented as the mean ± SEM of eight mice per group. ^#^ *p* < 0.05 vs. the control (CON) group; * *p* < 0.05, ** *p* < 0.01, and *** *p* < 0.001 vs. the HFD (60%)-induced obese group.

We next investigated whether EECM alleviated body weight gain and body fat accumulation in the tissues of HFD-induced obese mice. Body weight was considerably increased in the HFD group compared to the normal diet (CON) group during the 10-week administration period, whereas EECM administration significantly reduced the body weight gain from three weeks (Figure 4A). In addition, HFD-induced final body weight (HFD group: 42.24 ± 1.94 g vs. EECM 100 mg/kg group: 35.94 ± 0.83 g, *p* < 0.001; EECM 300 mg/kg group: 35.53 ± 1.06 g, *p* < 0.001) and weight gain (HFD group: 18.64 ± 0.44 g vs. EECM 100 mg/kg group: 12.99 ± 0.88 g, *p* < 0.001; EECM 300 mg/kg group: 12.91 ± 0.99 g, *p* < 0.001) were significantly attenuated by EECM treatment (Figure 4B,C).

### 3.5. EECM Changes the Fat Composition in HFD-Induced Obese Mice

**Figure 5:**
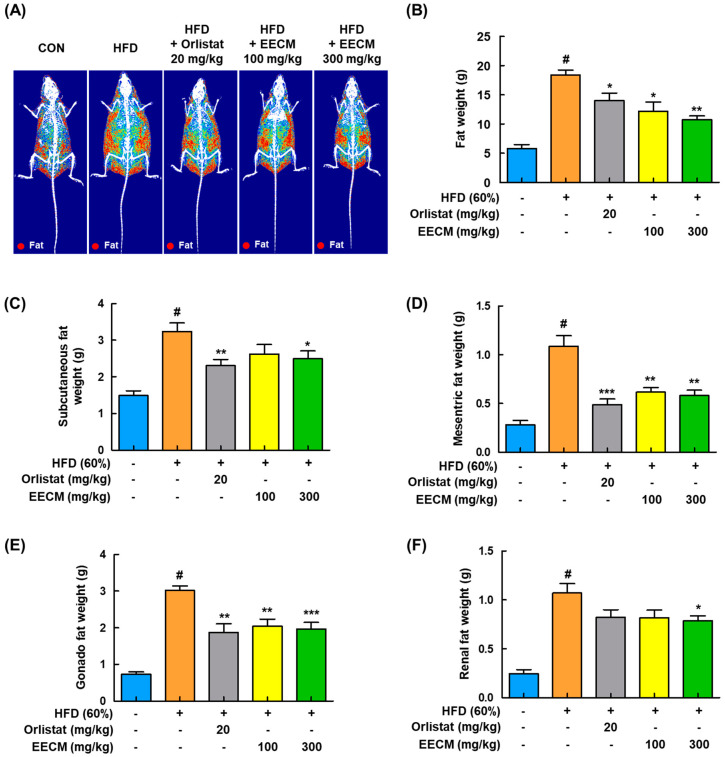
Regulatory effects of EECM on body composition and fat mass in HFD (60%)-induced obese mice. (**A**) Radiography images of the fat tissues are presented. (**B**) Fat weight in each group was measured via DEXA analysis. Fat was obtained from each tissue after the sacrifice of mice, and the weight was measured. (**C**–**F**) Subcutaneous fat, mesenteric fat, gonadal fat, and perirenal fat weights. Values are represented as the mean ± SEM (DEXA analysis; *n* = 3/group, fat in tissue weight: *n* = 8/group). ^#^ *p* < 0.05 vs. the CON group; * *p* < 0.05, ** *p* < 0.01, and *** *p* < 0.001 vs. the HFD (60%)-induced obese group.

For the identification of fat composition by EECM administration, we analyzed the body composition using DEXA in HFD-induced obese mice. As shown in Figure 5A,B, body fat accumulation was significantly induced in the HFD group compared to the CON group (CON group: 5.81 ± 0.68 g vs. HFD group: 18.38 ± 0.86 g, *p* < 0.05). On the contrary, EECM inhibited fat accumulation similarly to in the orlistat group, representing a positive control, and effectively improved the total body fat weight (HFD group: 18.38 ± 0.86 g vs. orlistat group: 13.97 ± 1.33 g, *p* < 0.05; EECM 100 mg/kg group: 12.20 ± 1.55 g, *p* < 0.05; EECM 300 mg/kg group: 10.73 ± 0.63 g, *p* < 0.01). In addition, EECM reduced the fat pad weight gain of subcutaneous, mesenteric, gonadal, and perirenal fat compared to that in the HFD group in a dose-dependent manner (Figure 5C–F), indicating that the daily supplementation of EECM can suppress body weight gain via the regulation of body fat composition.

### 3.6. EECM Alleviates the Production of Insulin, Adipokines, and Lipid Parameters in Blood Plasma of HFD-Induced Obese Mice

**Figure 6:**
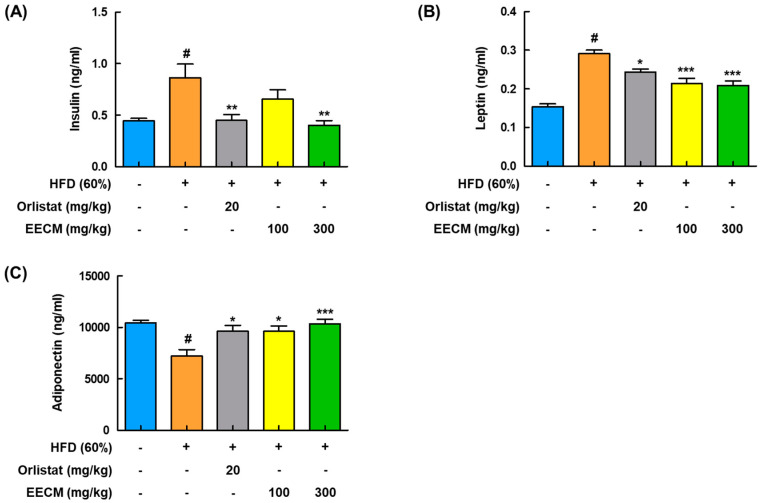
Regulatory effect of EECM on the production of adipocyte-released hormones in HFD (60%)-induced obese mice. Plasma levels of (**A**) insulin, (**B**) leptin, and (**C**) adiponectin were detected using ELISA kit. Values are represented as the mean ± SEM (*n* = 8/group). ^#^ *p* < 0.05 vs. the CON group; * *p* < 0.05, ** *p* < 0.01, and *** *p* < 0.001 vs. the HFD (60%)-induced obese group.

Next, to analyze the effects of EECM on the production of insulin and adipokines in plasma, including leptin and adiponectin, and lipid profiles in HFD-induced obese mice were investigated. HFD group had significantly higher levels of insulin and leptin than the CON group, whereas administration of EECM reduced those levels similarly to the orlistat group (Figure 6A,B). Levels of adiponectin decreased in the HFD group compared to those in the CON group but were significantly recovered by EECM treatment (Figure 6C). As shown in Table 1, EECM administration significantly ameliorated HFD-induced TC, TG, and LDL levels but had no significant effects on LDL and HDL levels.

### 3.7. EECM Prevents Hypertrophy of Lipid Droplets and Adipogenesis in Subcutaneous Fat of HFD-Induced Obese Mice

**Figure 7:**
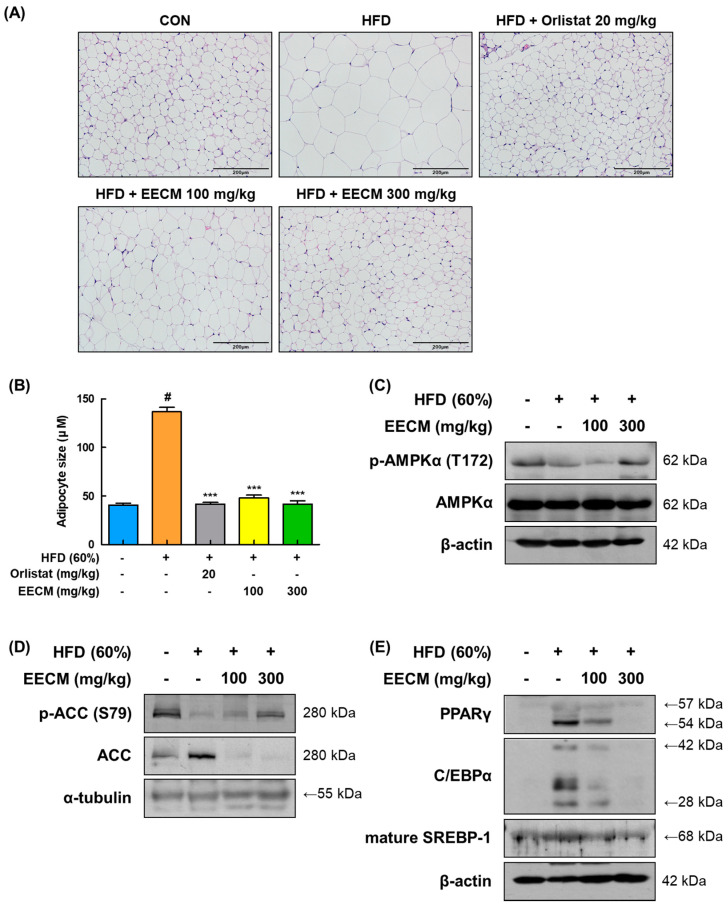
Effects of EECM on adipocyte size, adipogenesis, and lipogenesis in the subcutaneous fat tissues of HFD (60%)-induced obese mice. (**A**) Histological analysis of subcutaneous fat tissue via H&E staining at ×20 magnification. (**B**) Representative diameter of adipocytes in the tissue. Expression levels of (**C**) p-AMPKα, (**D**) lipogenic proteins, and (**E**) adipogenic transcription factors were determined via Western blotting analysis. Values are represented as the mean ± SEM (*n* = 8/group). ^#^ *p* < 0.05 vs. the CON group; *** *p* < 0.001 vs. the HFD (60%)-induced obese group.

We compared the histological construction of subcutaneous fat pads in each group using H&E staining. In the HFD group, lipid droplets of adipocytes were extremely enlarged in size compared to those in the CON group (CON group: 40.68 ± 1.78 μM vs. HFD group: 136.81 ± 4.50 μM, *p* < 0.001); conversely, EECM administration abated the magnification of adipocytes according to the increase in concentration (HFD group: 136.81 ± 4.50 μM vs. EECM 100 mg/kg group: 48.13 ± 2.67 μM, *p* < 0.001; EECM 300 mg/kg group: 41.70 ± 3.49 μM, *p* < 0.001; Figure 7A,B). Based on histological analysis, we verified the protein expression levels of adipogenesis-related proteins such as AMPKα, lipogenic enzymes, and adipogenic transcription factors. Phosphorylation of AMPKα (Thr172) was downregulated by HFD but restored by EECM administration (Figure 7C). Consistent with the in vitro data, EECM also upregulated the HFD-induced ACC phosphorylation (Ser79) (Figure 7D). Moreover, adipogenic factors including PPARγ, C/EBPα, and mature-SREBP-1 were expressed at higher levels in the HFD group than in the CON group, but EECM treatment lowered these levels (Figure 7E). Our data indicate the inhibitory effect of EECM on adipocyte hypertrophy is due to the regulation of adipogenesis-related proteins in the subcutaneous fat tissues of HFD-induced obese mice.

### 3.8. EECM Ameliorates Lipid Accumulation in Liver Tissue of HFD-Induced Obese Mice

**Figure 8:**
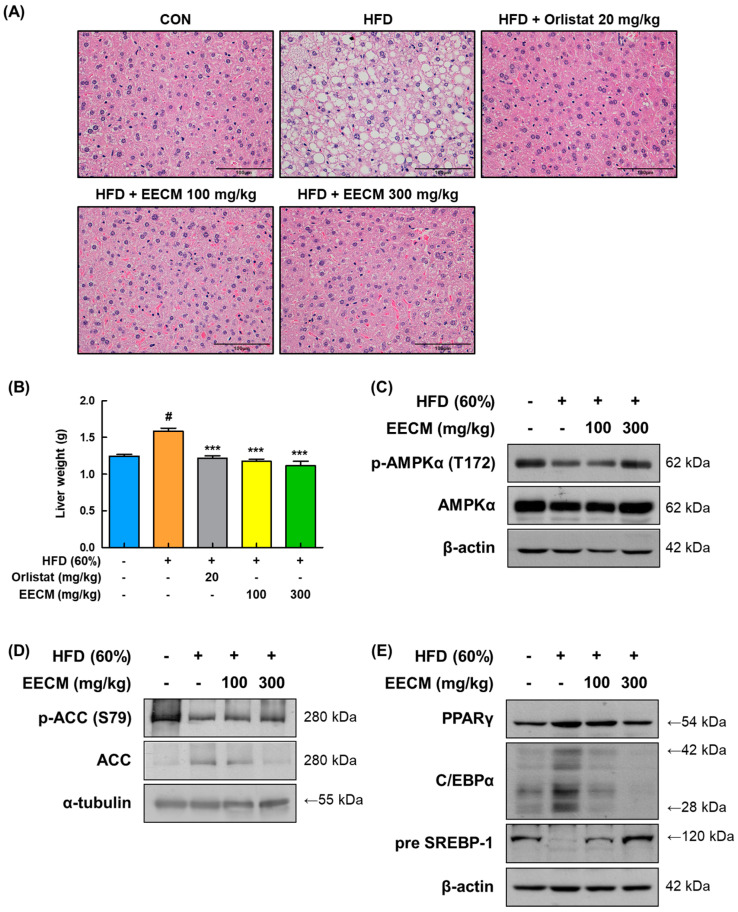
Effect of EECM on lipid accumulation in the liver tissues of HFD (60%)-induced obese mice. (**A**) The images of H&E stained the liver tissues at ×40 magnification. (**B**) Comparison of liver weights of each group. Expression levels of (**C**) p-AMPKα, (**D**) lipogenic proteins, and (**E**) adipogenic transcription factors were determined via Western blotting analysis. Values are represented as the mean ± SEM (*n* = 8/group). ^#^ *p* < 0.05 vs. the CON group; *** *p* < 0.001 vs. the HFD (60%)-induced obese group.

To verify the repressive effect of EECM on lipid accumulation leading to fatty liver, we performed H&E staining of the liver tissues of the animal model. As shown in Figure 8A, HFD induced lipid accumulation in the liver tissues, whereas EECM treatment restored this change remarkably and was similar to the positive control, the orlistat group. In addition, EECM significantly suppressed the weight gain of liver tissue in HFD-induced obese mice (HFD group: 1.59 ± 0.04 g vs. EECM 100 mg/kg group: 1.18 ± 0.03 g, *p* < 0.001; EECM 300 mg/kg group: 1.11 ± 0.06 g, *p* < 0.001; Figure 8B). In line with the results for subcutaneous fat, the levels of phospho-AMPKα (Thr172), phospho-ACC (Ser79), pre-SREBP-1, PPARγ, and C/EBPα were also downregulated following EECM administration (Figure 8C–E). These results indicate that EECM prevents liver steatosis by regulating the AMPK pathway as well as lipogenesis and adipogenesis in HFD-induced obese mice.

### 3.9. EECM Upregulates Thermogenic-Mediated Proteins Levels in Brown Adipose Tissue (BAT) of HFD-Induced Obese Mice

**Figure 9:**
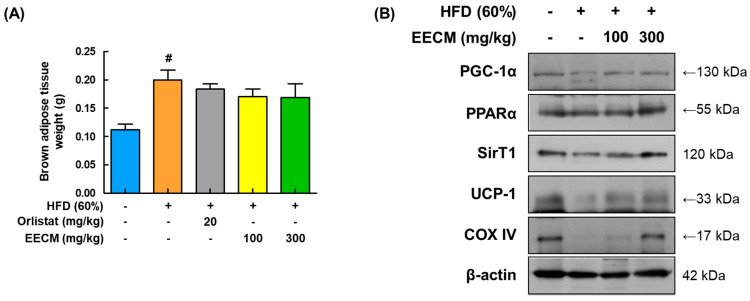
Stimulating effects of EECM on thermogenesis in the BAT. (**A**) Weights of BAT in each group. (**B**) Expression levels of thermogenesis-related proteins PGC-1α, PPARα, SIRT1, UCP-1, and COX-IV were determined via Western blotting analysis. Values are represented as the mean ± SEM (*n* = 8/group). ^#^ *p* < 0.05 vs. the CON group.

Mitochondrial thermogenesis in BAT provides protection against fat accumulation owing to its energy-dissipating activity [18]. After the mice were sacrificed, EECM administration slightly reduced the BAT weight compared to that in the HFD group (Figure 9A). Expression levels of major thermogenic-related factors, including PGC-1α, PPARα, SIRT1, and UCP-1, were markedly upregulated after EECM treatment compared to those in the HFD group. Additionally, EECM enhanced the protein levels of COX-IV and ATP synthase in the mitochondria (Figure 9B). These data imply that EECM-induced thermogenesis results in increased energy expenditure in the BAT of HFD-fed mice.

### 3.10. EECM Regulates the Gut Microbiota Composition and Alleviates the Dysbiosis in HFD-Induced Obese Mice

**Figure 10:**
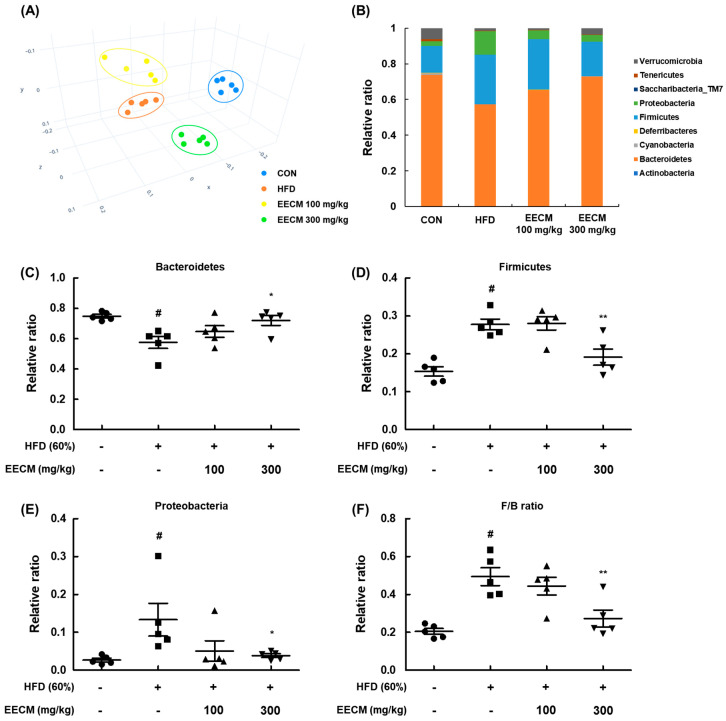
Regulatory effects of EECM on microbiota composition and colonization. (**A**) β-diversity between groups was analyzed using the principal coordinate analysis (PCoA) plot. (**B**) Ratio of phylum level composition ratio of each group. (**C**–**F**) Relative ratio of Bacteroidetes, Firmicutes, and Proteobacteria and the Firmicutes/Bacteroidetes (F/B) ratio. Values are represented as the mean ± SEM of five mice per group. ^#^ *p* < 0.05 vs. the CON group; * *p* < 0.05 and ** *p* < 0.01 vs. the HFD (60%)-induced obese group.

Finally, we evaluated the effects of EECM on the composition and colonization of gut microbiota in an animal model. To assess the variation in the phylogenetic relationship of microbial clusters between each group, we confirmed the principal coordinate analysis plot based on β-diversity. In the plot, the HFD group data were formed by shifting from the CON group community, and the groups treated with EECM were clustered away from the HFD group (Figure 10A). Next, we analyzed the phylum-level composition of the microbiome in each group (Figure 10B–E). HFD feeding increased the relative F/B ratio and decreased the relative ratio of Bacteroidetes, whereas EECM administration inversely regulated the ratio of each microbiota at the phylum level. Dysbiosis is frequently observed in obese individuals, and a large change in the F/B ratio is considered to be an indicator of dysbiosis [14]. Here, the F/B ratio significantly increased in the HFD group compared to that in the CON group, but EECM moderated this ratio compared to that in the HFD group (Figure 10F). These results indicate that EECM regulates the shift in gut microbiome composition and prevents dysbiosis in HFD-induced obese mice.

## 4. Discussion

Being overweight and obese are major risk factors for metabolic syndromes, CVD, T2D, and some cancer types [19]. According to the World Health Organization, the number of obese people nearly tripled between 1975 and 2016 [20]. Although there are various anti-obesity drugs, their limited effects and possible side effects demand a constant need to find novel and safe compounds for anti-obesity treatment. Natural-product-derived anti-obesity therapeutics are less toxic than synthetic drugs [21], and some drugs for obesity complications are derived from natural product materials [22]. *C. mimosoides* var. *nomame* Makino, a plant that grows naturally in Korea, Japan, and China, has the potential to be developed as a functional food material due to its scavenging of reactive oxygen species [15]. However, the methanol extract was not used because it is well-known that the toxicity of methanol is serious and cannot be used as an extraction solvent for development in health functional foods. Therefore, methanol extraction was excluded and ethanol used instead, as this experiment was designed to evaluate an efficacy study for the further development of health functional food. In the present study, EECM treatment not only resulted in a remarkable reduction in the number and diameter of 3T3-L1 preadipocytes during differentiation but also a decrease in body weight gain and fat weight in HFD-induced obese mice. Interestingly, as EECM administration did not affect the levels of GPT, GOT, and BUN in the blood plasma compared to those in the control or HFD groups (Figure S2), we believe that EECM does not cause considerable damage to liver and kidney function. Our data revealed EECM as a natural product with great potential for anti-obesity treatment. We further examined its target signaling activity in vitro and in vivo.

Insulin is a hormone secreted by pancreatic β cells, and the regulation of insulin clearance in the plasma is weak in obese subjects and patients with T2D [23]. Insulin concentration in the plasma of overweight individuals has been shown to correlate with plasma leptin levels [24]. Leptin, an adipokine secreted by adipocytes, is mainly associated with obesity, and the degree of leptin resistance in the blood plasma of most obese individuals is low [25]. Among the major adipokines, adiponectin plays an important role in obesity and influences weight loss; therefore, it has the potential to treat insulin resistance and obesity [26,27]. In this study, EECM administration downregulated the plasma levels of insulin and ameliorated the secretion of adipokines, including leptin and adiponectin. Furthermore, EECM administration decreased the plasma levels of lipid parameters including TC, TG, and LDL although it did not significantly affect the HDL levels. Therefore, our findings revealed that EECM mitigated dyslipidemia in an HFD-induced obese mouse model via the regulation of lipid profiles.

The early period of adipocyte differentiation involves complex signaling including adipogenesis, lipogenesis, and cell cycle regulation [28]. PPARγ and C/EBPα are the main regulators of adipogenesis that are involved in adipocyte differentiation [29]. PPARγ expression is induced during the proliferation and differentiation of adipocytes, and it promotes intracellular lipid accumulation [30]. In addition, the cross-regulation between PPARγ and C/EBPα is important for the transcriptional regulation of adipogenesis [31]. SREBP-1 is present as an ectopic protein of the pre-form (inactive), which binds SREBP cleavage-activating protein to the complex in the endoplasmic reticulum (ER) membrane, and the mature form (active), which is cleaved and released from the ER membrane [32]. Mature-SREBP-1 enters the nucleus and sustains lipogenesis in adipocytes at the transcriptional level [33,34]. Phosphorylation (Thr 172) of AMPK inhibits adipogenesis, fatty acid storage, and lipogenesis by increasing the activity of related factors, such as PPARγ, C/EBPα, and SREBP-1 [35]. Activated AMPK is associated with the regulation of lipogenic enzymes including ACC, a major regulatory factor in sterol and lipid synthesis [36], and FAS [37]. ACC catalyzes the conversion of acetyl-CoA to malonyl-CoA following the phosphorylation of ACC (inactive form) by AMPK, and the production of malonyl-CoA is reduced, eventually resulting in fatty acid oxidation [38]. In the present study, EECM exerted anti-obesity effects in vitro and in vivo by suppressing the expression of PPARγ, C/EBPα, SREBP-1, and FAS and p-ACC expression via the AMPK pathway.

During early adipocyte differentiation, MCE is tightly regulated by cell cycle regulators and differentiation-related factors prior to terminal differentiation [39]. The proliferation of adipocytes is stopped, and the cells enter the MCE process by treatment with MDI, a differentiation inducer [40,41]. Our results showed that treatment with EECM increased the protein expression levels of cyclin D1, cyclin E1, cyclin B1, CDK6, and CDK2 in MDI-differentiated 3T3-L1 pre-adipocytes. Moreover, EECM treatment restored cell cycle progression from the G2/M phase. In addition, the protein expression of p21, a CDK inhibitor that regulates G1/S phase progression [42], was increased by EECM treatment although the recovery level was weak. Therefore, EECM suppressed MCE during the early differentiation of preadipocytes.

Among primary adipose tissues, BAT plays a significant role in energy expenditure and thermogenesis [10]. Energy dissipation occurs in mitochondria and leads to ATP generation, which further increases heat production [43]. UCP-1, the major thermogenic regulator of the mitochondrial inner membrane, causes heat generation by uncoupling respiration from ATP production [44]. In addition, the mitochondrial biomarker, COX IV, is a major regulator of oxidative phosphorylation that mediates the final step of the electron transfer chain in mitochondria [43]. PGC-1α induces the expression of thermogenic genes, including the *PPAR* family and *UCP-1*, via an adaptive process for energy dissipation, resulting in the lipolysis of cellular TG [45,46]. AMPK increases cellular NAD^+^ levels and activates SIRT-1 (NAD^+^-dependent deacetylase), resulting in PGC-1α activation and upregulation of adiponectin expression [47]. Therefore, hepatic lipid accumulation can be mitigated via the AMPK/SIRT1 pathway [48]. In this study, we found that the administration of EECM increased the protein expression of thermogenesis-related factors, including PGC-1α, PPARα, UCP-1, and COX IV, and the activation of AMPKα, which induced the expression of SIRT1. Based on these results, we believe that the modulating effect of EECM on thermogenesis in BAT may affect lipolysis in HFD-induced obese mice.

Several studies have reported that the composition and colonization of the gut microbiome are related to various dietary and pathophysiological changes such as obesity [49,50,51,52]. In general, the gut microbiome in the human intestine has a dominant F/B ratio (approximately 90%) [53]. Proteobacteria is often reported in diseases such as obesity, NAFLD, and metabolic syndrome [7]. Previous studies reported that the proportion of Firmicutes was higher than that of Bacteroidetes in the obese colon, whereas the opposite was observed in the lean state [17,54]. Accordingly, the F/B ratio is considered as one of the measures to analyze obesity and dysbiosis [14]. In this study, we observed that the composition of the microbiome was altered in HFD-induced obese mice compared to that in the normal-diet group, and it was recovered by EECM administration. Moreover, EECM also reduced the F/B ratio compared to that in the HFD group, indicating its anti-obesity effect.

## 5. Conclusions

In summary, our results revealed that EECM ameliorates obesity in vitro and in vivo. Treatment with EECM suppressed MCE and differentiation of 3T3-L1 preadipocytes and downregulated the protein expression levels of adipogenesis-related markers. In the subcutaneous fat and liver of HFD-induced obese mice, oral administration of EECM significantly decreased the body weight gain and lipid accumulation and suppressed the protein expression of adipogenic factors. In addition, EECM activated thermogenesis in BAT by upregulating the expression levels of thermogenic proteins. EECM also restored gut microbiota dysbiosis and blood levels of insulin, leptin, and adiponectin in HFD-induced obese mice. Taken together, our findings confirm the anti-obesity effects of EECM and highlight its potential to be used as a functional food ingredient from natural products.

## Figures and Tables

**Table 1:** Effects of the ethanol extract of *Cassia mimosoides* var. *nomame* Makino (EECM) on the lipid parameters in HFD (60%)-induced obese mice.

Lipid Profiles	CON	HFD	Orlistat (20 mg/kg)	EECM (100 mg/kg)	EECM (300 mg/kg)
TC (mg/dL)	104.88 ± 5.08	157.00 ± 3.14 ^#^	130.25 ± 13.19 **	145.00 ± 6.54	142.38 ± 4.04 *
TG (mg/dL)	55.50 ± 4.18	112.25 ± 5.87 ^#^	67.75 ± 8.35 **	70.00 ± 9.14 **	63.00 ± 9.58 ***
LDL (mg/dL)	10.13 ± 0.81	15.00 ± 0.50 ^#^	11.88 ± 0.91 *	12.63 ± 0.53	12.63 ± 0.46
HDL (mg/dL)	85.75 ± 2.10	92.88 ± 4.97	113.13 ± 9.27	102.25 ± 3.22	100.50 ± 2.05

Values are represented as the mean ± standard error of the mean (SEM) (*n* = 8/group). ^#^ *p* < 0.05 vs. the CON group; * *p* < 0.05, ** *p* < 0.01, and *** *p* < 0.001 vs. the HFD (60%)-induced obese group.

## Data Availability

Not applicable.

## Supplementary Materials

The following supporting information can be downloaded at: https://www.mdpi.com/article/10.3390/nu15030613/s1, Figure S1: Cytotoxicity of EECM in 3T3-L1; Figure S2: Hepatoxicity and nephrotoxicity of EECM in the blood plasma of HFD-induced obese mice; Figure S3: Representative area of adipocytes in the tissue; Figures S4–S8: Quantitative values for Western blot in Figure 2, Figure 3, Figure 7, Figure 8 and Figure 9.
