# Supplementary material for: Standardized Ethanol Extract of Cassia mimosoides var. nomame Makino Ameliorates Obesity via Regulation of Adipogenesis and Lipogenesis in 3T3-L1 Cells and High-Fat Diet-Induced Obese Mice
Source: Nutrients. 2023 Jan 25;15(3):613. doi: 10.3390/nu15030613 (PMC9920205; doi:10.3390/nu15030613)
Supplement: Supplementary file 1 [file nutrients-15-00613-s001.zip › nutrients-2159302-supplementary.pdf]

Supplementary Materials

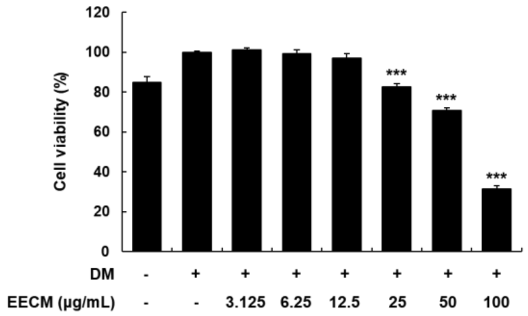

Figure S1. The 3T3-L1 cells were cultured with GM for 3 days, the media was replaced by DM with MDI and various concentration of EECM (3.125-100 μg/mL) was treated until 7 days. After differentiation, an MTS assay was performed to measure cell viability. Values are represented as the mean ± SD. *** $p < 0.001$.

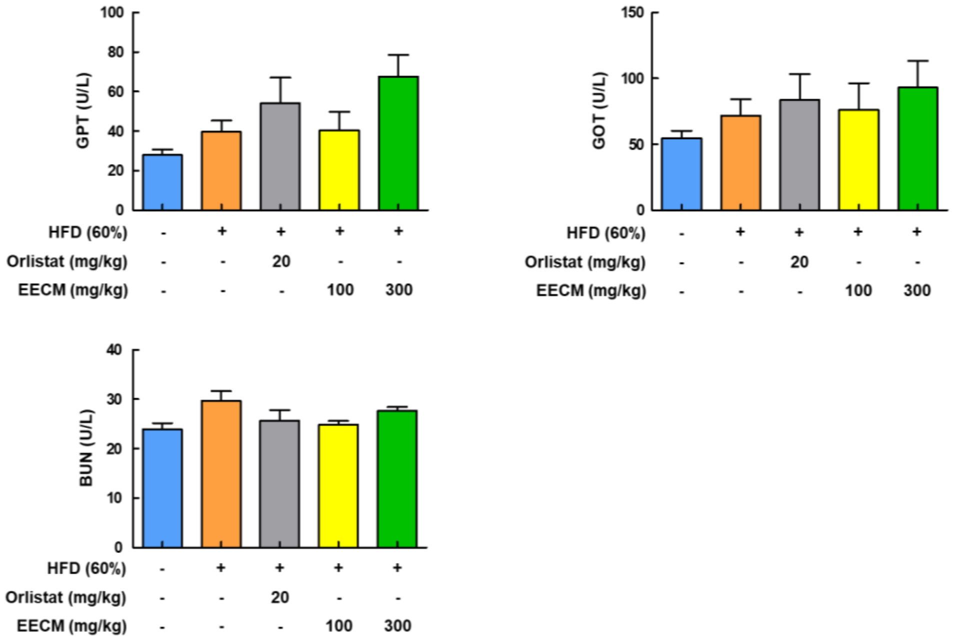

Figure S2. The blood plasma levels of glutamic oxaloacetate transaminase (GOT), glutamate pyruvate transaminase (GPT), and blood urea nitrogen (BUN).

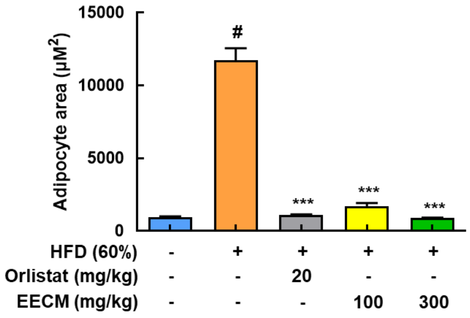

Figure S3. Representative area of adipocytes in the tissue. Values are represented as the mean ± SEM. $^{\#}p < 0.05$ vs. the control group; *** $p < 0.001$ vs. the HFD-induced obese group.

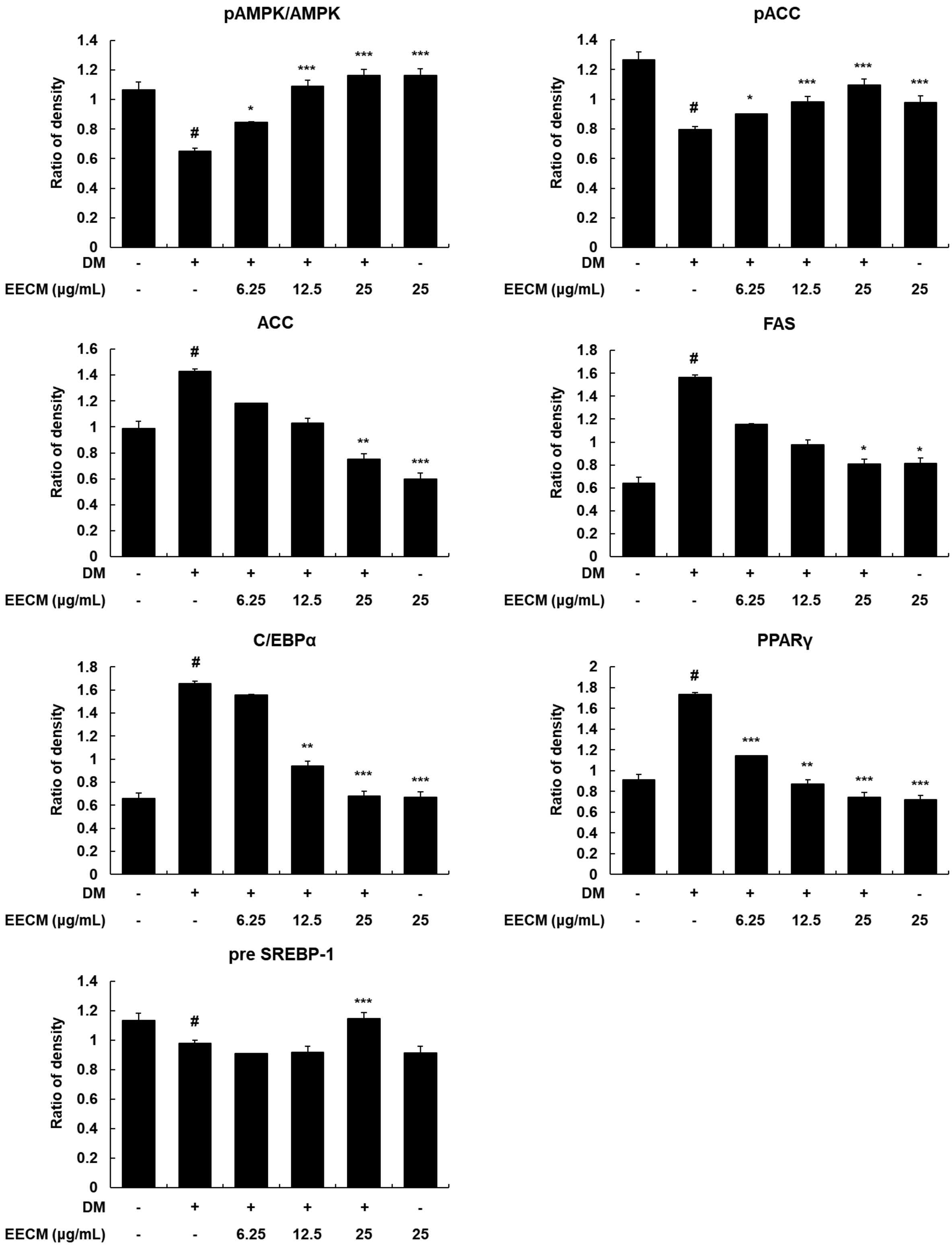

Figure S4. Quantitative values for Western blot in Figure 2. Values are represented as the mean ± SD. $^{\#}p < 0.05$ vs. the GM group; $^{*}p < 0.05$, $^{**}p < 0.01$, and $^{***}p < 0.001$ vs. the DM group.

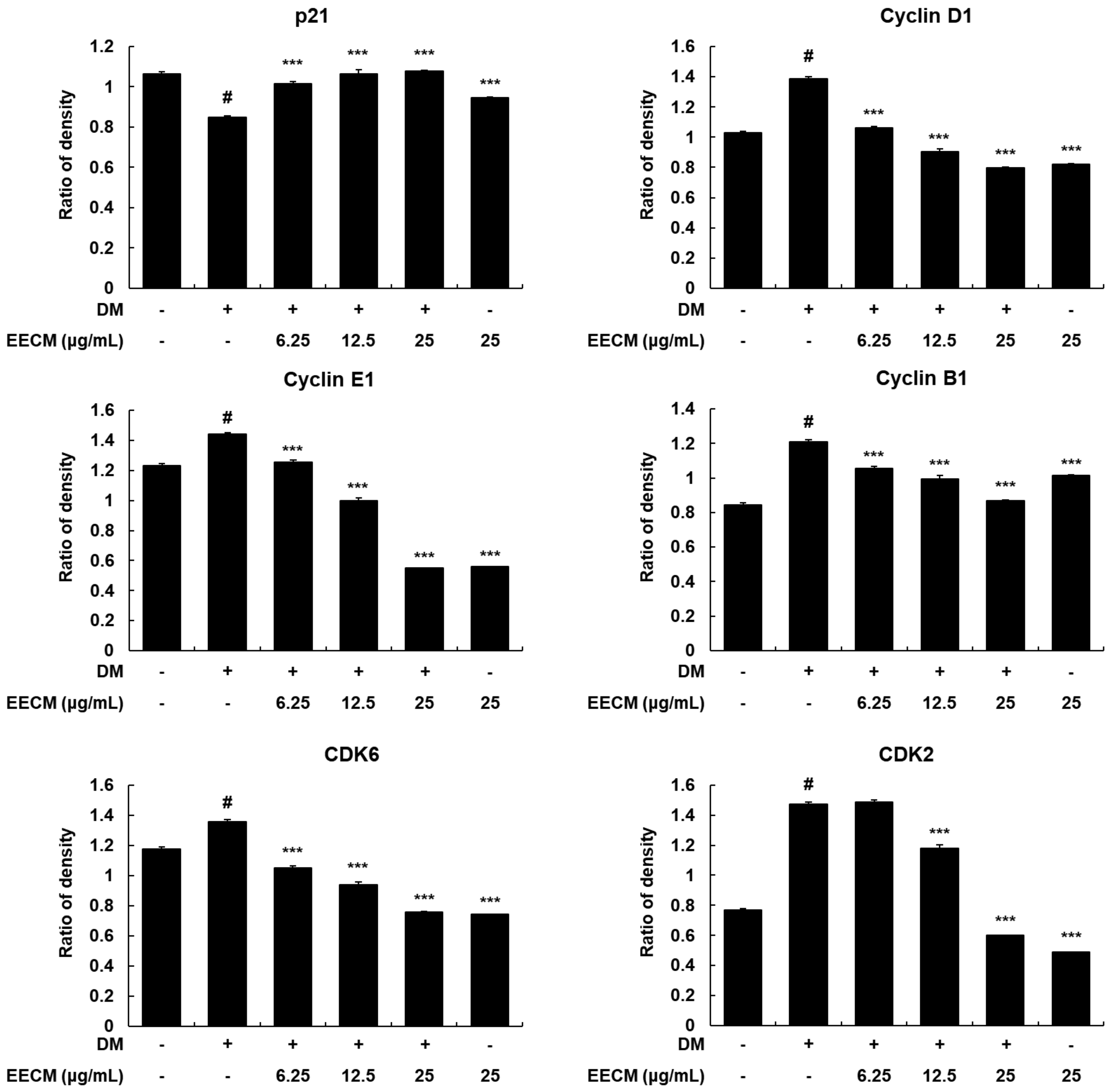

Figure S5. Quantitative values for Western blot in Figure 3. Values are represented as the mean ± SD. $^{\#}p < 0.05$ vs. the GM group; $^{***}p < 0.001$ vs. the DM group.

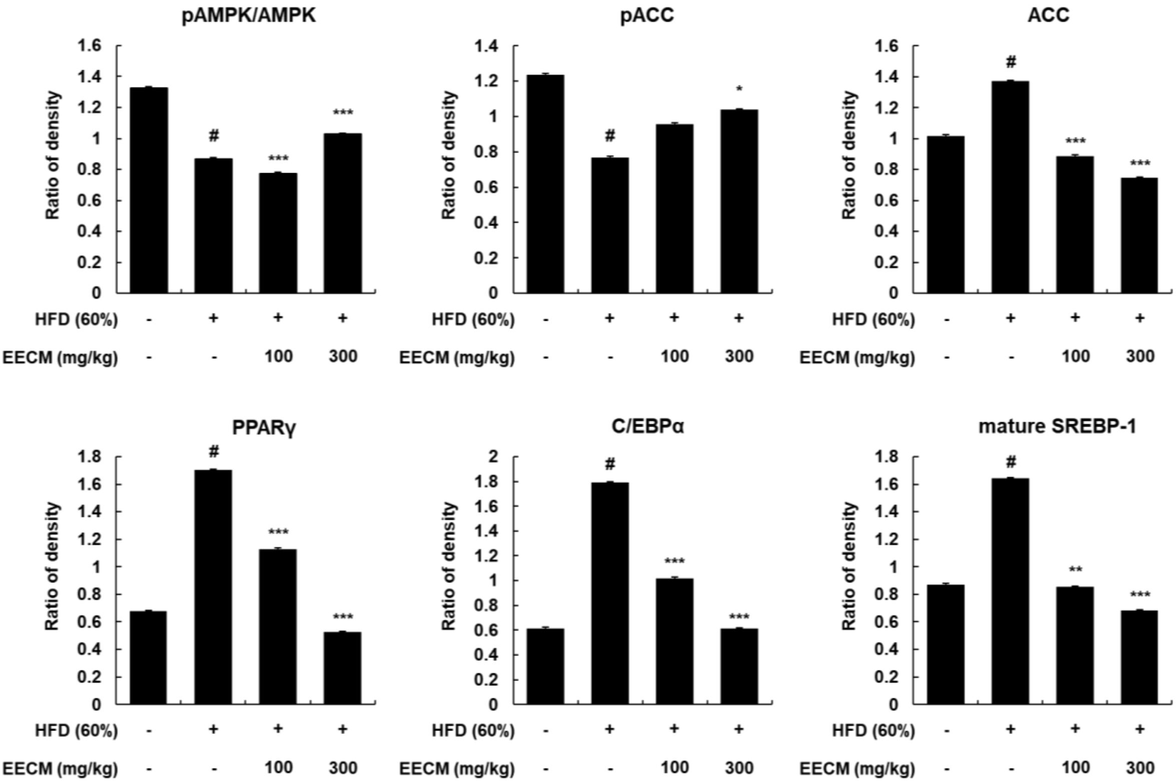

Figure S6. Quantitative values for Western blot in Figure 7. Values are represented as the mean ± SEM. $^{\#}p < 0.05$ vs. the control group; $^{*}p < 0.05$, $^{**}p < 0.01$, and $^{***}p < 0.001$ vs. the HFD-induced obese group.

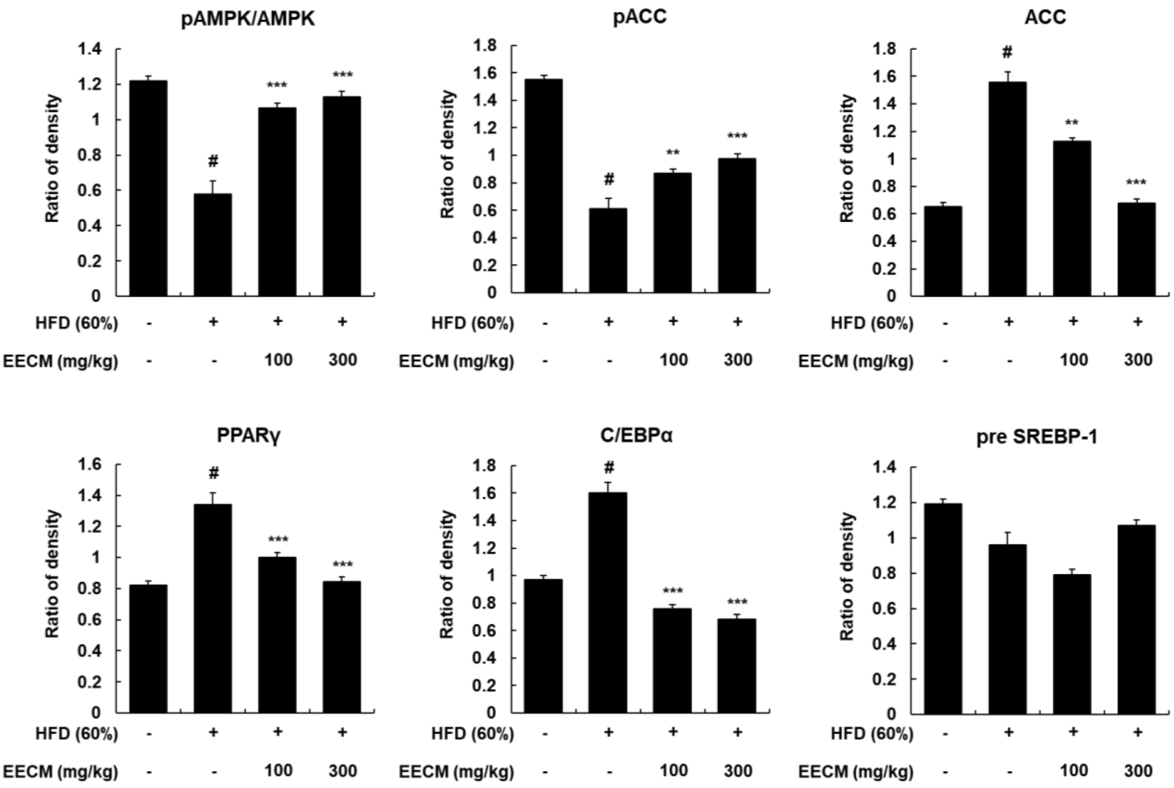

Figure S7. Quantitative values for Western blot in Figure 8. Values are represented as the mean ± SEM. $^{\#}p < 0.05$ vs. the control group; $^{**}p < 0.01$, and $^{***}p < 0.001$ vs. the HFD-induced obese group.

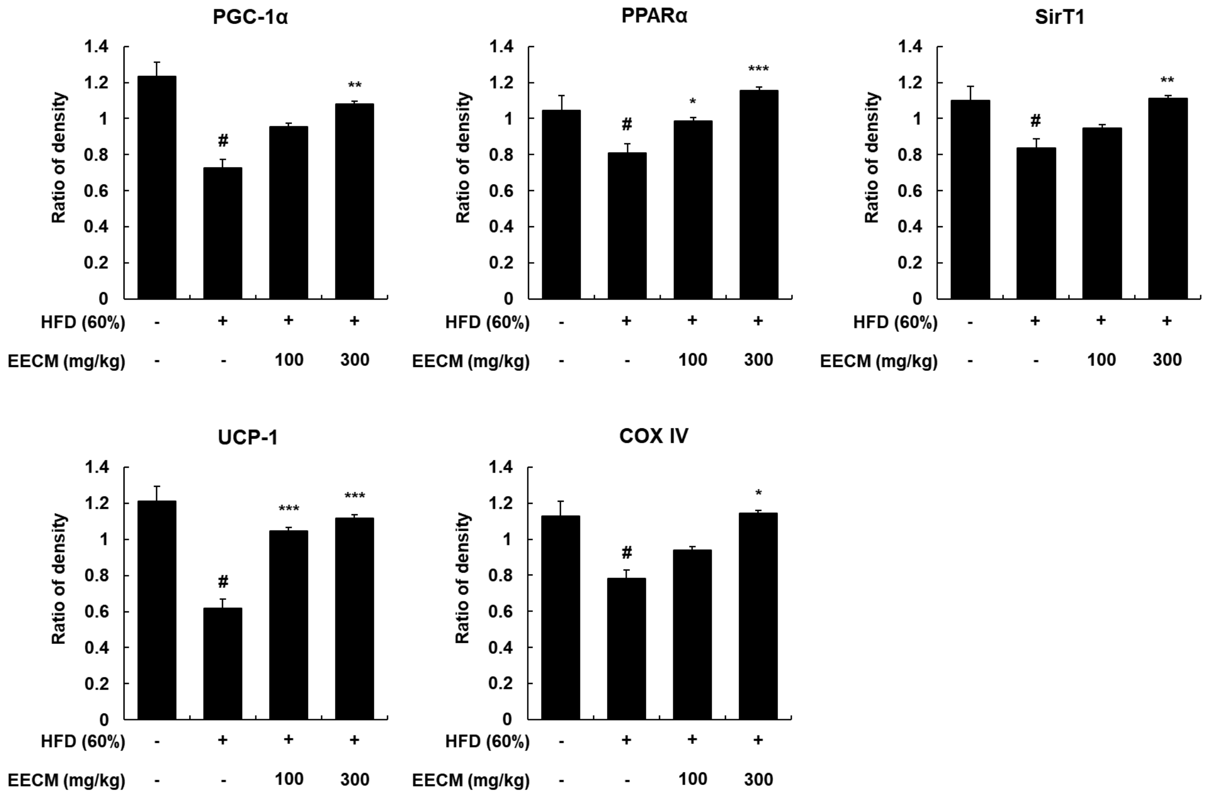

Figure S8. Quantitative values for Western blot in Figure 9. Values are represented as the mean ± SEM. $^{\#}p < 0.05$ vs. the control group; $^{*}p < 0.05$, $^{**}p < 0.01$, and $^{***}p < 0.001$ vs. the HFD-induced obese group.
